# A retrospective study of reducing unnecessary thyroid biopsy for American College of Radiology Thyroid Imaging Reporting and Data Systems 4 assessment through applying shear wave elastography

**DOI:** 10.20945/2359-3997000000267

**Published:** 2020-07-17

**Authors:** Jieli Luo, Jianshe Chen, Yang Sun, Fangting Xu, Lilu Wu, Pintong Huang

**Affiliations:** 1 Second Affiliated Hospital Zhejiang University School of Medicine China Department of Ultrasound, the Second Affiliated Hospital of Zhejiang University School of Medicine , Hangzhou, China

**Keywords:** Shear-wave elastography, thyroid nodule, American College of Radiology Thyroid Imaging Reporting and Data Systems, fine needle aspiration biopsy

## Abstract

**Objective:**

The purpose of the study is to quantitatively assess shear-wave elastography (SWE) value in American College of Radiology Thyroid Imaging Reporting and Data Systems (ACR TI-RADS) 4.

**Materials and methods:**

One hundred and fifty-two ACR TI-RADS 4 thyroid nodules undergoing SWE were included in the study. The mean (EMean), minimum (EMin) and maximum (EMax) of SWE elasticity were measured.

**Results:**

The areas under the receiver operating characteristic (ROC) curves for SWE EMean, EMin and EMax in detecting benign and malignant nodules were 0.95, 0.83 and 0.84, respectively. Cut-off value of EMean ≤ 23.30 kPa is able to downgrade the lesion category to ACR TI-RADS 3 and cut-off value of EMean ≥ 52.14 kPa is able to upgrade the lesion category to ACR TI-RADS 5.

**Conclusions:**

The EMean of SWE will probably identify nodules that have a high potential for benignity in ACR TI-RADS 4. It may help identify and select benign nodules while reducing unnecessary biopsy of benign thyroid nodules.

## INTRODUCTION

Thyroid nodules are common findings in the general population and have increased in recent years ( 1 - 3 ). Most thyroid nodules are actually asymptomatic and benign, and only about 5% of thyroid nodules are malignant ( 4 - 8 ). Ultrasound is the most accurate way to image thyroid nodules because malignant ultrasound features are associated with a higher malignancy risk ( 9 ). But no ultrasound feature has both high sensitivity and high specificity ( 10 ). According to the guidelines, fine needle aspiration biopsy (FNAB) is the best way to identify benign and malignant tumors ( 11 - 13 ). But FNAB also has some limitations, such as being invasive and time consuming. Elastography, a new imaging technique, is a non-invasive procedure that is simple and convenient, the least but not the last, its capability of stiffness quantification as additional diagnostic metric, was added to the diagnostic list to evaluate thyroid nodules ( 14 ).

Several Thyroid Imaging Reporting and Data Systems (TI-RADS) have been developed for malignancy risk stratification that incorporate ultrasound features to categorize thyroid nodules, such as French TI-RADS, American Thyroid Association (ATA) guideline, Korean TI-RADS and American College of Radiology Thyroid Imaging Reporting and Data Systems (ACR TI-RADS) ( 15 - 19 ). The ACR TI-RADS developed a predictive model that assigned different risk scores to each suspicious ultrasound feature and determined the TI-RADS category of nodules by allocating points to more suspicious features and summing the total score ( 20 - 23 ). ACR TI-RADS 3 assessment assesses lesions with a malignancy of less than 5% and ACR TI-RADS 4 assessment for lesions with a malignant risk, ranging from 5% to 20% ( 24 ).

Ultrasound strain elastography measures the deformation of tissue responsiveness and display and derive its stiffness. The disadvantage of this kind ultrasound elastography is that diagnostic accuracy and quality of elastography may be affected by internal compression (e.g., depth and distance of carotid artery pulsation, depth of nodule to skin, tracheal motion) and external compression ( 25 ). However, shear wave elastography (SWE) estimates the shear wave velocity of the tissue by acoustic radiation force of the ultrasonic push pulses and directly reflects the tissue stiffness with Young’s modulus ( 26 ). Qualitative and quantitative assessments of tissue stiffness can be obtained ( 27 , 28 ). However, the nodule stiffness measurement can be biased by the pre-load due to the weight of the transducer and the operator’s hand over-compressing superficial and thyroid tissue. Fortunately, this can be avoided by using a thick gel layer. SWE is the most reproducible and least operator-dependent technique among other elastographic techniques ( 29 ).

## MATERIALS AND METHODS

### Patient selection

The patient cohort was retrospectively collected from patients assessed from January 2017 to May 2018. Informed consent was obtained from all patients. Most patients were selected for FNAB based on ACR TI-RADS, or because they were at high risk based on history, such as a family history of thyroid cancer or a history of radiation exposure. A very small part of patients were strongly recommended FNAB by themselves.

All examinations were performed by the same operator with 5 years of ultrasound experience. Patients with ACR-TIRADS 4 nodules who underwent FNAB or surgery after routine ultrasound and SWE were included in the study. Exclusion criteria were as follows: 1. Nodule with rim calcification or macrocalcification; 2. Nodule with cystic ingredient; 3. Nodule with depth > 3 cm; 4. Thyrodititis. SWE was performed after gray-scale ultrasound and prior to the FNAB.

### Ultrasound examination

Patient was in the supine position with slightly extension of the neck on the examination table. After application of coupling agent, both conventional ultrasound imaging and SWE were performed with Mindray Resona 7 ultrasound machine equipped with high frequency (5.6 ∼ 10 MHz) linear array transducer before FNAB. Conventional ultrasound scanning protocols included both transverse and longitudinal imaging of thyroid nodules. Two thyroid radiologist with 7 years of clinical experience evaluated category according to ACR TI-RADS who were blinded to each other. Two radiologists agreed on the form of the discussion if there was disagreement between them. SWE images of longitudinally oriented thyroid nodules were obtained from the same two sonographers who underwent elastography training for more than 5 years. The transducer was placing lightly on the patient’s neck and it was necessary to make sure that the neck was not under pressure. Patients were asked to keep a breath for a while to minimize the effect of breathing when the sonographer collected standard images. The qualified images were as follows: 1. The color signal frame was almost completely filled with color and also the color was stable. 2. There was no obvious compression artifact. Analyzer was blind to the cytological result when placed region of interest (ROI) over the entire nodule. Choosing the highest stiffness region of each nodule, each ROI was recorded by the mean (EMean), min (EMin) and max (EMax) stiffness values of SWE.

### ACR TI-RADS

**Figure 1:**
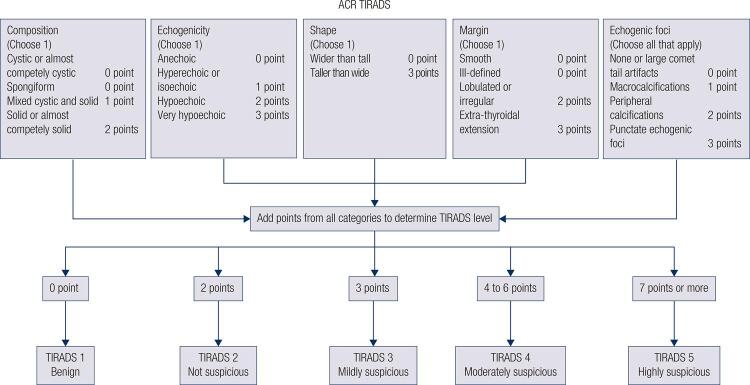
Schematic diagram of ACR TI-RADS was showing in the figure.

According to five features categories in the ACR-TIRADS Lexicon (shape, margin, composition, echogenicity, echogenic foci), the examiner assigned a malignancy risk that matched the five risk stratification levels (benign, not suspicious, mildly suspicious, moderately suspicious or highly suspicious) ( Figure 1 ). We elaborate on the most misunderstanding ultrasound findings about the five groups. A spongiform nodule which is included in composition must be composed predominantly (> 50%) of small cystic spaces. The feature of echogenicity uses the adjacent thyroid tissue as the basis for comparison. Except for very hypoechoic nodules, in which the echogenicity refers to a nodule’s reflectivity relative to the strap muscles. Taller-than-wide was categorized as when the anteroposterior diameter of a nodule was longer than its transverse diameter on a transverse or longitudinal plane for nodule shape. Microcalcification was defined as calcification with a diameter less than 1 mm, which was visualized as tiny and punctiform. Intra- and inter-observer reproducibility of ACR-TIRADS categories was assessed.

### Final diagnosis

The final diagnosis was determined by histopathology or follow-up of each thyroid nodule. FNAB result was categorized as six grades. Bethesda category II to III was defined as benign and Bethesda category IV to VI was defined as malignant. For benign thyroid nodules, the final diagnosis was confirmed by surgery, repeated FNAB at least two benign outcomes, or one-time benign result on FNAB, and no change size on follow-up ultrasound more than 12-month. For malignant thyroid nodules, the final diagnosis was confirmed by surgery or a malignant result on FNAB at once.

### Statistical analyses

All statistical analyses were conducted with IBM SPSS Statistics for Windows 22.0 version (Chicago) and Graph Pad Prism 6. Intra- and inter-observer reproducibility were tested in all cases using Kappa values. Comparing the velocities of benign and malignant lesions was used by the Mann-Whitney U test. The difference of measurement data was detected by Kolmogorov-Smirnov test. “rule out” and “rule in” elastography value was assessed by estimating the area under the receiver operating characteristic (ROC) curve. A sensitivity of at least 98% was considered a requirement to downgrade a lesion (“rule-out criterion”). A specificity of at least 98% was considered a requirement to upgrade a lesion (“rule-in criterion”). P value less than 0.05 considered statistically significant.

## RESULTS

**Figure 2:**
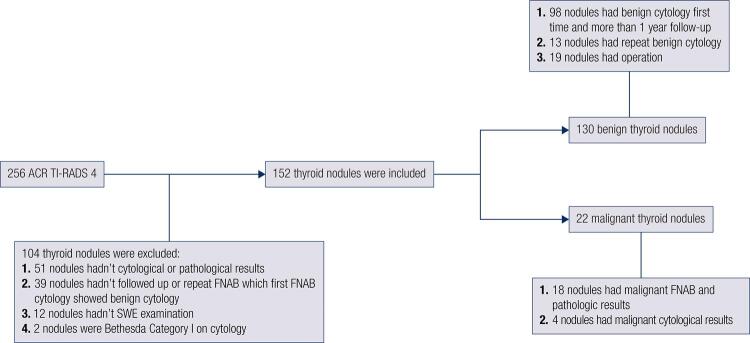
Two hundred and fifty-six ACR TI-RADS 4 lesions were evaluated in the initial analysis. One hundred and four lesions were excluded for some reasons as shown in figure. The remaining 152 lesions were used for the final analysis.

Two hundred and fifty-six lesions were included in the initial analysis from January 2017 to May 2018 with an ACR TI-RADS score of 4. One hundred and four lesions were eliminated from this analysis for some certain reasons. Definitive diagnosis was obtained via biopsy or surgical excision for 152 thyroid lesions form 152 patients. The mean age ± SD of the 152 patients (112 men and 40 women) were 45.2 ± 12.4 years (range, 19-74 years). Result revealed 22 malignant nodules and 130 benign nodules (including 37 benign nodules comfirmed by radiological follow-up and the remaining comfirmed by pathological results). The specific flow chart of thyroid lesion was described in figure 2 . The total thyroid cancer detection rate was 14.5% in our subjects. There were no significant differences in age and nodule diameter between malignant and benign groups ( Table 1 ). The Kappa values of intra- and inter-observer reproducibility of ACR-TIRADS categories were 0.81 and 0.77.

**Table 1:** Patient demographics

Characteristic	Value		P value

	Benign nodule	Malignant nodule	
Number, n (%)	130 (85.5%)	22 (14.5%)	< 0.05
Age, year (mean ± SD)	45.88 ± 12.47	41.36 ± 11.95	> 0.05
Gender, n (%)			
Female	30	10	< 0.05
Male	100	12	< 0.05
Longest diameter (cm)	1.08	1.12	> 0.05
SWE, Emin (kPa)	9.80	19.67	< 0.05
SWE, Emean (kPa)	24.15	47.37	< 0.05
SWE, Emax (kPa)	57.86	92.07	< 0.05

SWE: shave wave elastography.

**Figure 3:**
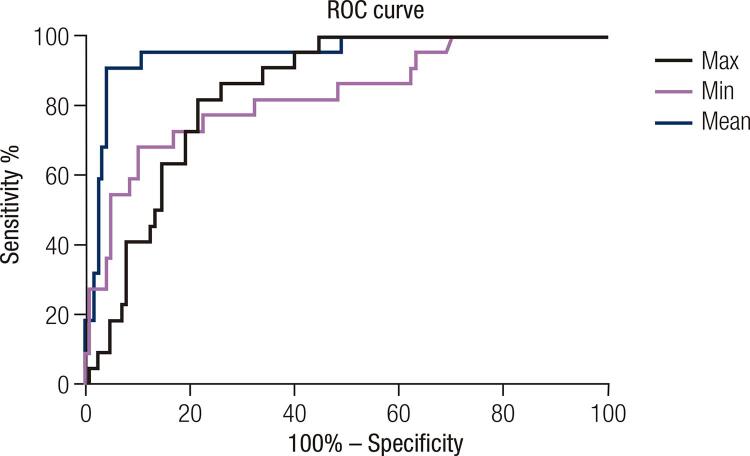
The SWE value of mean (EMean), min (EMin) and max (EMax) with areas under the receiver operative characteristic (ROC) curve of 0.95, 0.83 and 0.84, respectively.

**Figure 4:**
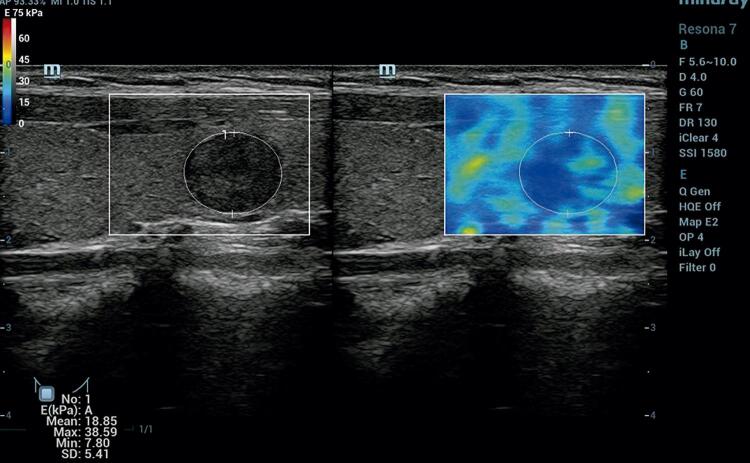
According to composition (solid), echogenicity (very hypoechoic), shape (wider than tall), margin (smooth) and echogenic foci (none), this nodule was ACR TI-RADS 4 with 5 points. The EMean of SWE was 18.85 kPa which was able to downgrade the lesion category to ACR TI-RADS 3. The pathology of this nodule was nodular hyperplasia.

The area under the ROC curve value of EMean, EMin and EMax were 0.95 (95% CI 0.91-1.00), 0.83 (95% CI 0.73-0.93), 0.84 (95% CI 0.78-0.91) respectively ( Figure 3 ). EMean yielded the highest area under the ROC curve value, which was used for the diagnosis of nodules. Cut-off value of EMean ≤ 23.30 kPa was able to downgrade the lesion category (“rule-out criterion”) to ACR TI-RADS 3 with a sensitivity > 98%, Cut-off value of EMean ≥ 52.14 kPa was able to upgrade the lesion category (“rule-in criterion”) to ACR TI-RADS 5 with a specificity > 98% ( Figure 4 ).

## DISCUSSION

Previous studies had focused on the overall study of thyroid nodules at all levels, while rare reports had discussed ACR TI-RADs 4 nodules individually ( 30 ). However, in clinical work, it was the ACR TI-RADS 4 nodule that made the examining physician more confused ( 31 ). In particular, it was more difficult to conduct a good assessment, and it was impossible to provide meaningful guidance to clinicians when there was no experience ( 32 ).

Routine detection thyroid nodules used by high-frequency ultrasound couldn’t detect the biophysical parameters closely related to the above-mentioned tumor tissues. The measurements of traditional ultrasound elastography technology were affected by probe pressure and the operator’s subjective experience, which led to unsatisfactory diagnostic specificity and sensitivity to benign and malignant thyroid nodules. SWE technology measured the speed of shear waves emitted by transducers in different tissues and evaluates nodules with Young’s modulus values ( 33 ). It was the only technique that could quantitatively determine the stiffiness value of nodules at present ( 34 ). SWE had achieved “sound and palpation” with strong objectivity, good repeatability and little influence by the operator.

Benign thyroid lesions were mainly consisted of follicular cells, which were filled with colloid components and were soft in texture, while the intratumoral cancer cells replacing the soft follicles and glial components in malignant thyroid lesions ( 35 - 37 ). The increased in the stiffiness of malignant thyroid lesion was due to the fact that the tissue mainly contains many interstitial fibrous tissues, blood vessels and concentrically arranged calcified bodies and other reasons. In addition, the increased in the stiffiness of papillary thyroid cancer was related to the formation of a large number of psammoma bodies within the nodule.

SWE like other technologies had a certain false positive and negative rate. Overlapping of lesion information was because when some benign nodules develop chronic fibrosis and coarse calcification during growth, the stiffiness increased accordingly and also EMax value increased, resulting in false positives; Szczepanek-Parulska and cols. also reported that benign lesions with microcalcifications were stiffer than benign lesions without calcifications, complicating the use of maximal SWE stiffness to discriminate benign and malignant lesions ( 38 ). On the other hand, malignant nodules decrease stiffness due to necrosis, liquefaction, and hemorrhage and also EMax values decreased, resulting in false negatives. It was difficult to distinguish with benign nodules in certain situation. EMean yielded the highest area under the ROC curve value, which was the best indicator used for the diagnosis of nodules. Cut-off value of EMean ≤ 23.30 kPa was able to downgrade the lesion category (“rule-out criterion”) with a sensitivity > 98%, Cut-off value of EMean ≥ 52.14 kPa was able to upgrade the lesion category (“rule-in criterion”) with a specificity > 98%. The easily accessible and reliable SWE will complement the ACR TI-RADS 4, support clinical decision making and reduce necessary thyroid biopsy.

There were some limitations in this study. First, there was a retrospective and single study, a selection bias was inevitable. Second, stiffness value measurements only achieved by longitudinal images. We favored of longitudinal orientation due to the lower incidence of images affected by carotid pulsation as well as lack of tracheal compression on thyroid. Third, operator-dependent factors (lack of contact of probe with neck, blurring caused by probe motion and so on) of SWE were not excluded in this study.

Our study support the recommendation that ACR-TIRADS 4 combined with SWE could be used in clinical diagnosis as a noninvasive diagnostic approach, which may lead to a great improvement in accurate diagnosis, and the number of unnecessary FANB could be reduced for benign thyroid nodules.
